# Nuclear Fragmentation Imaging for Carbon-Ion Radiation Therapy Monitoring: an In Silico Study

**DOI:** 10.14338/IJPT-20-00040.1

**Published:** 2021-09-01

**Authors:** Anissa Bey, Jiasen Ma, Keith M. Furutani, Michael G. Herman, Jedediah E. Johnson, Robert L. Foote, Chris J. Beltran

**Affiliations:** 1 Department of Radiation Oncology, Mayo Clinic, Rochester, MN, USA; 2 Department of Radiation Oncology, Mayo Clinic, Jacksonville, FL, USA

**Keywords:** carbon radiation therapy, pencil-beam scanning, treatment monitoring, dose profile, beam range

## Abstract

**Purpose:**

This article presents an in vivo imaging technique based on nuclear fragmentation of carbon ions in irradiated tissues for potential real-time monitoring of carbon-ion radiation therapy (CIRT) treatment delivery and quality assurance purposes in clinical settings.

**Materials and Methods:**

A proof-of-concept imaging and monitoring system (IMS) was devised to implement the technique. Monte Carlo simulations were performed for a prospective pencil-beam scanning CIRT nozzle. The development IMS benchmark considered a 5×5-cm^2^ pixelated charged-particle detector stack positioned downstream from a target phantom and list-mode data acquisition. The abundance and production origins, that is, vertices, of the detected fragments were studied. Fragment trajectories were approximated by straight lines and a beam back-projection algorithm was built to reconstruct the vertices. The spatial distribution of the vertices was then used to determine plan relevant markers.

**Results:**

The IMS technique was applied for a simulated CIRT case, a primary brain tumor. Four treatment plan monitoring markers were conclusively recovered: a depth dose distribution correlated profile, ion beam range, treatment target boundaries, and the beam spot position. Promising millimeter-scale (3-mm, ≤10% uncertainty) beam range and submillimeter (≤0.6-mm precision for shifts <3 cm) beam spot position verification accuracies were obtained for typical therapeutic energies between 150 and 290 MeV/u.

**Conclusions:**

This work demonstrated a viable online monitoring technique for CIRT treatment delivery. The method's strong advantage is that it requires few signal inputs (position and timing), which can be simultaneously acquired with readily available technology. Future investigations will probe the technique's applicability to motion-sensitive organ sites and patient tissue heterogeneities. In-beam measurements with candidate detector-acquisition systems are ultimately essential to validate the IMS benchmark performance and subsequent deployment in the clinic.

## Introduction

Owing to a denser ionization track (high linear energy transfer [LET]) and enhanced relative biological effectiveness (RBE) [1], carbon-ion radiation therapy (CIRT) is an increasingly promising modality for treating radioresistant, hypoxic, and recurrent tumors [2, 3]. Mounting evidence suggests that low LET proton and photon radiation therapies lead to modest control of such malignancies. An inherent drawback of conventional photon therapy is the exposure of healthy tissues before and beyond the clinical target volume (CTV). This pitfall stems from the attenuation profile of photon beams in the body: peak dose deposition in the first few centimeters of tissue and exponential decrease thereafter. In contrast, on account of the characteristic Bragg peak (BP), the energy deposition of therapeutic charged particle beams (proton, carbon) exhibits a sharp fall-off beyond the CTV [3]. The steeper beam distal edge in particle therapy may allow more precise tumor targeting [4, 5] and has the potential to enable dose escalation treatment paradigms with improved critical organ sparing [6]. Moreover, carbon ions have less lateral scattering and energy straggling than protons [7]. The properties of CIRT were shown to support treatment effectiveness allied to the reduction of late toxicity [8, 9].

Despite the biological and physical advantages of carbon ions, the typical dose gradients delivered in CIRT pose distinct challenges for treatment delivery accuracy and quality assurance. One significant source of uncertainty stems from determining carbon ions' stopping powers in heterogeneous patient anatomy and water [4], which is typically used in treatment planning simulations. Carbon ions encounter varying anatomical composition and density along the beam travel path to reach the CTV. The dose distribution is sensitive to changes in patient anatomy (eg, tumor volume shrinkage, organ motion, changes in external contours due to changes in weight) and positioning. Hence, individualized in vivo determination of the beam range is needed to achieve more precise treatment delivery. With such capability, targeting subcentimeter accuracy [10] as well as better use of the steep dose fall-off in CIRT could be attainable [11].

Real-time monitoring of CIRT is therefore highly recommended (1) to ensure accurate (below the clinically acceptable 5% variability threshold [4, 12]) dose delivery to the patient and (2) to enable corrective action in fractionated treatment regimens. “Real-time monitoring” refers here to obtaining instantaneous and actionable monitoring information while the patient is irradiated on the treatment couch.

**Figure 1.**
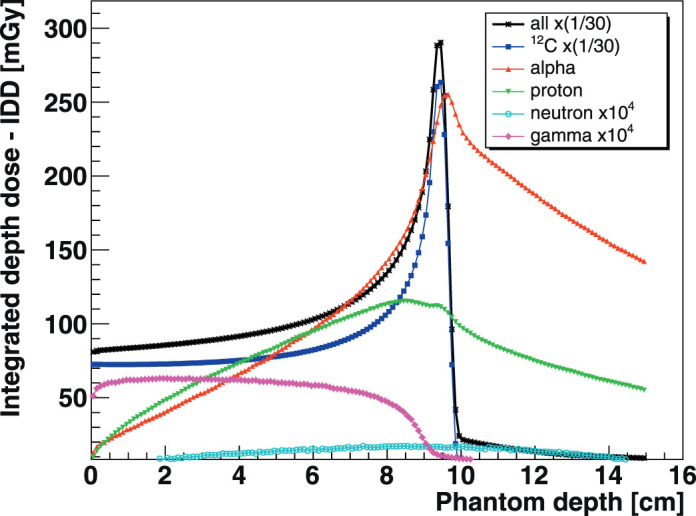
Simulated Bragg curve for a 220-MeV/u (WET = 10.3 cm [13]) carbon ion beam impinging on a 15×15×15-cm^3^ water phantom using 10^6^ histories. Individual dose contributions are delineated for the dominant secondary charged fragments: protons, ^4^He, and indirectly ionizing neutral radiations (M.G.H., oral communication, 2020). Individual dose scaling factors were applied for visibility. Abbreviation: WET, water equivalent thickness.

In CIRT, beam energies on the order of 90 to 290 MeV/u are required to penetrate a water equivalent thickness (WET) of 2.1 to 16.4 cm [13]. The ablation-abrasion reaction process was proposed to describe heavy-ion target collisions at these intermediate energies [14, 15]. Nuclear reactions of the ^12^C projectile with traversed tissues produce a mixed secondary radiation field formed by neutrons, prompt γ, e^−^/e^+^, and energetic charged fragments. The secondary charged radiation field is dominated by projectilelike fragments: H, He, Li, Be, B isotopes, in order of abundance. These fragments carry much of the ^12^C projectile's momentum, range longer in tissues (Z < 6), form forward peaked secondary beams, and may escape the CTV. The lightest charged fragments, hydrogen and ^4^He, account for the bulk of the dose tail beyond the CIRT BP as shown in **Figure 1** (M.G.H., oral communication, 2020).

Trace activities of the ^10,11^C and ^15^O β^^+^^ emitters are also produced in more peripheral ^12^C-tissue (^12^C, ^16^O) interactions, which spurred interest in in vivo position emission tomography (PET) monitoring of CIRT. In-room PET imaging solutions were developed and reported by several working groups [16–19]. While CIRT-PET enables beam range verification, notable technique limitations include the following: (1) reconstructed PET activity uncorrelated to the delivered dose profile since the two arise from different processes, (2) activity physiological washout in the patient, (3) requirement of high geometric detection coverage to compensate for low activities, and (4) complex and often cumbersome in-room equipment.

Charged fragment–based real-time monitoring overcomes these limitations with appreciably higher production cross sections and detection efficiency. As noted earlier, the secondary charged fragments produced in CIRT may escape tissues, preserving information about their parentage. In previous conceptual studies [20, 21], the interaction vertex imaging (IVI) technique was applied to secondary proton detection. Proton trajectories were used to reconstruct the interaction vertices, that is, the points where these particles originated along the beam path. The reconstructed vertex distribution was shown to inform on the delivered depth dose curve.

We investigated the performance of an IVI monitoring system (IMS) for a prospective active spot scanning CIRT (AS-CIRT) delivery nozzle at our institution. Special focus was placed on identifying persistent dosimetry and monitoring markers for online, in vivo plan delivery verification.

In the clinical workflow, planning computed tomography scans are often acquired several days before the start of treatment, increasing the susceptibility of the plan to patient anatomical changes. The IMS should, therefore, allow for real-time monitoring of a CIRT treatment delivery. Such control could enable beam delivery interruption in the event that a threshold-exceeding divergence arises between the planned and real-time measured value of a monitored marker.

The incentives of the present study were to recognize technology limitations and cost-effectiveness as key factors when designing a prospective detector system solution. The system's underlying goal is to achieve stable and persistent monitoring in clinical settings. From the detection feasibility standpoint, a simple and fast-response monitoring system would require (1) minimal signal processing, relinquishing expensive transformations such as pulse height analysis; (2) no particle-type discrimination or identification, removing the need for sophisticated equipment to measure energy loss and/or time of flight; and (3) a flexible data acquisition format in lieu of binned frame mode imaging.

In this first conceptual IMS study, we show that CIRT fragment identification is not necessary for dose delivery verification, augmenting the work of Henriquet et al [20], which assumed hypothetical particle discrimination.

## Materials and Methods

The IMS proof-of-concept was investigated for a brain CIRT case: glioblastoma multiforme, a primary brain tumor with markedly poor prognosis and hallmarks of radioresistance [22, 23].

### Simulations Framework

Monte Carlo (MC) simulations were used to generate the IMS detected signals. These signals were analyzed in a manner emulating the response of a realistic detector system to infer potential plan relevant monitoring markers.

#### Monte Carlo simulation code

The TOPAS MC code version 3.2 [24], back-ended by the Geant4 toolkit's 10.5 release [25], was used to perform efficient simulations of the AS-CIRT delivery nozzle and IMS assembly. Special attention was dedicated to select relevant physics interactions for modeling the complex secondary radiation field of incident carbon ions. For the hadron-nuclear inelastic part, the built-in Binary IntraNuclear Cascade (BIC) and Quantum Molecular Dynamic (QMD) models were compared for fragment yields and angular and energy distributions, with focus on the dominant isotopes. The BIC model, whose performance was benchmarked against datasets measured at 95, 200, 310, and 400 MeV/u [26, 15, 20, and 27 and 28, respectively], was chosen as a compromise on the ground of avoiding excessive overestimation of yields. While the in-field dose due to neutrons is negligible, their energies span orders of magnitude and are tracked down to thermal energies. Hence, the data-driven high precision (NeutronHP) neutron package was used to transport neutrons below 20 MeV. Hadron elastic scatterings were modeled with the G4HadronElasticPhysicsHP package. Electromagnetic interactions used the G4 Standard EM option 4 physics list.

#### CIRT nozzle and beam source

**Figure 2.**
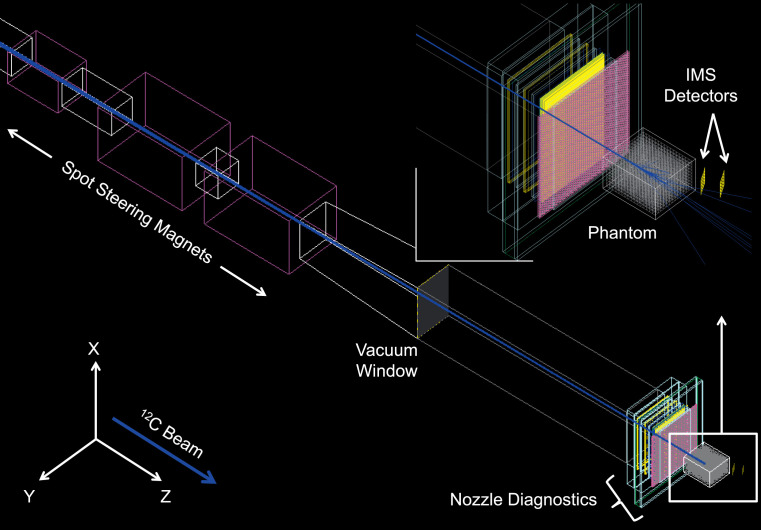
Geometric layout of the AS-CIRT delivery nozzle, head phantom, and IMS detector tracker implemented in the MC simulations. Abbreviations: AS-CIRT, active spot scanning–carbon-ion radiation therapy; IMS, imaging and monitoring system; MC, Monte Carlo.

Detailed MC simulations of prospective AS-CIRT nozzle designs were completed in a separate study and will be reported in a future work. The investigated nozzle system (**Figure 2**) transported the carbon ion beam in air and consisted of horizontal (Y) and 2 vertical (X) spot steering dipole magnets, vacuum section pipes and windows, and a diagnostics block. The latter is composed of a beam spot scanning monitor (SPM), a multi-wire proportional gas chamber, dose monitors, and an energy modulation “mini-ridge” filter.

The carbon beam phase-space was sampled from a Twiss Gaussian source, with emittance parameters provided by the vendor. For a 20-cm nozzle tip clearance, the AS-CIRT pencil-beam focal spot size was σ__X__: 1.3 mm × σ__Y__:1.6 mm in air at the isocenter. The beam energy straggling was 0.96% at 220 MeV/u (WET = 10.3 cm). The spread-out Bragg peak width for 1 energy layer at 220 MeV/u in the studied phantom was 2.8 (± 0.2) mm. All simulations considered 10^6^ histories per scanned beam spot.

#### Phantom and target

A voxelized geometric phantom (X:15.35, Y:10.65, Z:18.05 cm^3^) was implemented to represent a human head. The phantom was modeled as grey matter brain tissue (voxel size of 1 mm^3^) surrounded by a 6.75-mm-thick cortical bone shell.

The elemental weights and mass densities of these anatomical materials were taken from Schneider et al [29]. The clinical target to be externally spot scanned was assumed to be a glioblastoma multiforme tumor mass with approximately the same tissue composition (37 HU [Hounsfield units]) as the brain grey matter (37–45 HU) [30].

#### Beam range

In this work, the beam range was determined at the BP 75% to 70% mark dose fall-off from the integrated depth dose distribution. The range was defined at the middle of the finite resolution voxel.

### Detector and Data Acquisition System

The CIRT charged fragments were detected in a 5×5-cm^2^, 50-μm-thick position-sensitive detector stack formed by a front and back trackers spaced by 5 cm. The device spatial resolution was set to an achievable 55-μm pixel pitch (eg, [31, 32]), while maintaining a conservatively small active read-out area. Silicon, a semiconductor commonly used in charged particle detection, was used as the detector material. The Si-stack was positioned 14 cm downstream from the center of the head phantom (nozzle isocenter) at a 30° angle relative to the primary carbon ion beam axis. This orientation was chosen to achieve an optimal trade-off between fragment statistics (largest at 0°) and limiting the reconstruction uncertainties induced by the beam finite spot size (smallest at 90°), as discussed in Piersanti et al [33].

An alternative IMS setup consisting of 2 detector stacks positioned downstream from the phantom at +30°/−30° with reference to the beam axis and operated in coincidence was also investigated in this work.

The technique's prerequisite is list-mode data acquisition. The read-out signal stream was strictly limited to 3 outputs for further processing and analysis: event identifier (ID), hit time stamp (t), and hit pixel (XYZ), that is, spatial coordinates. The fragment trajectories were constructed by requiring a front-to-back detector coincidence within a preset timing gate window on a stack and event-by-event basis. The trajectories of the primary beam ions were taken from the diagnostics SPM considering a 5×5-cm^2^ active area around the central axis.

### Reconstruction Algorithm

A beam axis back-projection principle method was implemented to reconstruct the reaction fragments' production points, that is, vertices. The hardness of the dominant fragments' (^1,2,3^H, ^3,4^He) energy spectra (eg, medians of 104 MeV/u for protons and 85 MeV/u for ^4^He at E(^12^C) = 240 MeV/u, WET = 11.9 cm) suggests the latter may scatter relatively minimally in tissues as they exit the target, thus allowing their trajectories to be reasonably approximated by 3D lines. The interaction vertex was defined as the point of closest approach (PCA) or minimal Cartesian distance between the fragment and beam ion trajectories.

A geometric reconstruction algorithm, written in C++, analyzed the detected events stream to determine the PCA as follows. First, fragment trajectories were calculated from coincidence timing between the trackers in the stack. Second, the fragment and beam hits were correlated, if both hits were registered within the same acquisition event ID. The optimization problem's cost function entailed the squared projected distances of the fragment and beam lines onto the PCA point. ^T^hird, unregularized least square minimization of the cost function computed the probable PCA solution.

## Results

### Reconstructed Vertices

**Figure 3.**
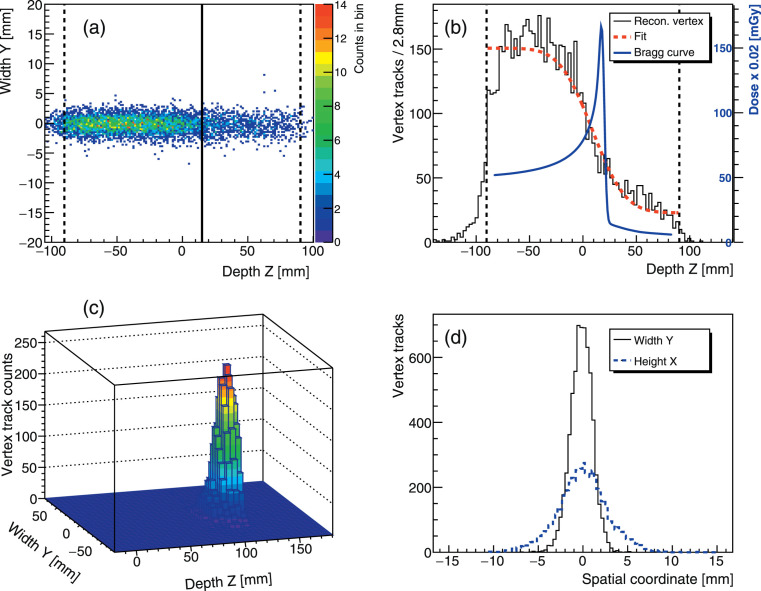
(a) Reconstructed 2D fragment vertex distribution at E(^12^C) = 240 MeV/u, range = 10.52 cm (solid vertical line), represented in the head phantom lateral (Y) and relative depth (Z) coordinates. The zero value on the z-axis indicates the center of the phantom located at 9.025 cm from its entrance. The dashed vertical lines mark the phantom boundaries. (b) Projected 1D vertex depth distribution with the overlaid Bragg curve and analytical fit. (c) Lego plot representation of data in (a) in absolute vertex depth coordinate. (d) Overlaid 1D vertex lateral (Y) and longitudinal (X) distributions.

The reconstructed vertex yield distributions are shown in **Figure 3** for the detected charged fragments at a 240-MeV/u beam energy (10.52-cm beam range in the studied phantom), simulating a target centered at 1.5 cm downstream from the middle (depth Z = 9.025 cm) of the head. Assuming a 100% detection efficiency, the dominant fragments detected in the front tracker amount to 1.6% of the transmitted beam events, a statistics that is further reduced by a 47% intrastack, that is, front-back, coincidence efficiency.

The MC data were exploited independently to verify and interpret the IMS reconstructed events. To illustrate the secondary radiation field produced in CIRT, at 240 MeV/u, about 50% of proton signals originated from the primary ^12^C beam vertex, suggesting secondary particle (proton, neutron)–induced reaction productions. Protons emanating from secondary vertices generated detector hit multiplicities of 2 and 3 and triggered uncertainties in the reconstruction algorithm. The ^4^He fragments on the other hand were dominantly primary (∼90%) in origin (compared to ∼50% for ^3^He), consistent with the prompt 3-α cluster break-up of ^12^C, which exhibits a threshold energy at 7.275 MeV. Furthermore, nearly 30% of detected ^4^He event vertices stemmed from out-of-field interactions of the beam with the nozzle's diagnostics.

### Monitoring Markers

#### Phantom boundaries and patient positioning

The phantom boundaries coincide with the rise and fall-off of the reconstructed vertex depth distributions. The broadening of the emission distribution (**Figure 3a**) can be attributed to light particles (eg, protons) that scatter while leaving the target and to those particle tracks created away from the primary beam axis. As explained in “Detector and Data Acquisition System,” the beam finite spot size (a Gaussian shape) was not considered ad hoc in the reconstruction procedure.

#### Dose depth profile and beam range

The projected vertex distribution along the phantom depth correlates with the delivered dose profile, represented by the overlaid Bragg curve for reference (**Figure 3b**). The expected CIRT dose fall-off is clearly discernible, as illustrated for a 240-MeV/u irradiation, and qualitatively corresponds to the primary ion beam range. The range to vertex fall-off correlation holds at the studied therapeutic energies. Strikingly, the BP location in the phantom is similarly recreated from a Lego plot of the vertex depth and width coordinates (**Figure 3c**).

To quantify the ion range correlation, an analytical parametrization (**Equation 1**) based on the complementary error function (*erfc*) [20] was used to fit the vertex depth distribution:



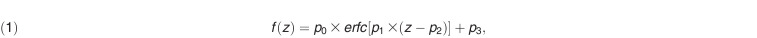

where *z* is the phantom depth; *p_i_*, *i* = 0-3, are free parameters to be determined in the fitting procedure; and *p*_3_ represents the inflection point of *erfc*.

In the in silico benchmark study we used to develop the IMS, which considered a water phantom (15-cm depth), the average difference between the vertex-profile–fitted inflection point and actual beam range was below 1.5 cm. The regions of best agreement were observed near the middle of the phantom. We considered that the inflection point did not correlate directly, in sufficient precision, with the beam range.

**Figure 4.**
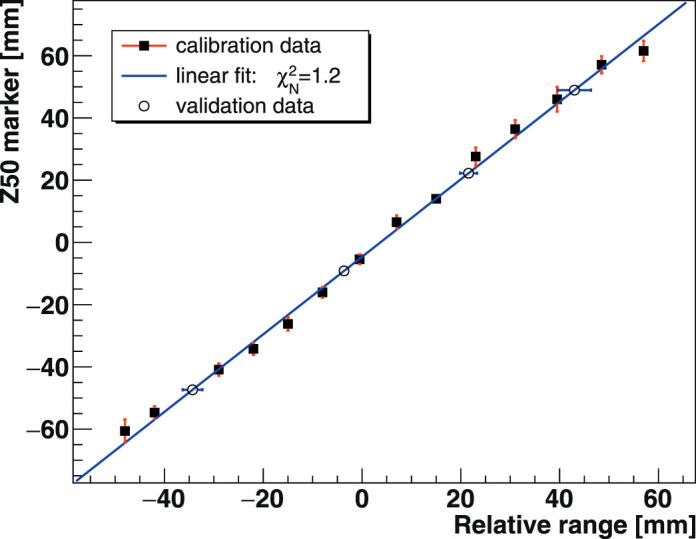
Beam range calibration using the Z50 dosimetry marker performed for irradiation energies between 150 and 290 MeV/u.

To improve the ion range verification accuracy, a depth marker “Z50” was calculated instead. This maker is defined at the depth corresponding to 50% of the maximum height of the fitted vertex depth distribution. A direct linear relationship was found between Z50 and the ion beam range. **Figure 4** reports the beam range calibration as a function of Z50 performed for incident energies between 150 and 290 MeV/u, spanning ranges of 4.22 to 14.72 cm.

The beam range and Z50 marker are expressed relative to the phantom depth, that is, the zero value on the z-axis corresponds to the middle of the phantom. A linear fit to the data produces the following formula with a fit goodness χ^2^/*ndf* = 1.2 (*ndf*, number of degrees of freedom):



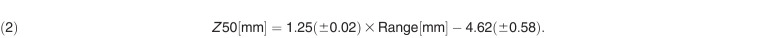




To mimic CIRT online monitoring where Z50 is extracted for each spot-scanned position, **Equation 2** was applied to a pseudo validation set obtained at irradiation energies between 160 and 280 MeV/u, which were not used in the calibration procedure. The beam range was determined within 3-mm accuracy, with an uncertainty ≤ 10% across the studied tissue depths.

#### Beam spot position

**Figure 5.**
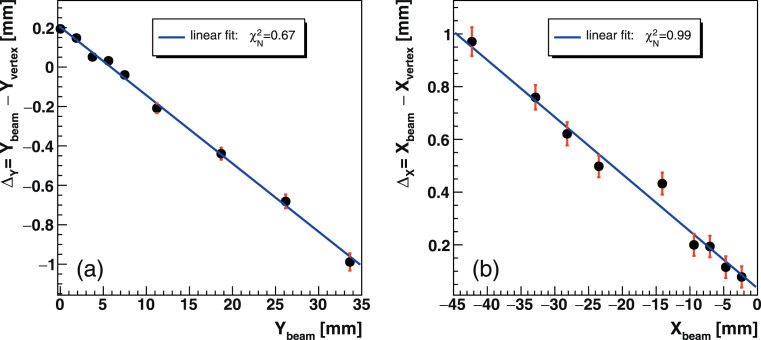
Beam spot position verification accuracy for the (a) lateral and (b) vertical directions in the head phantom at 220 MeV/u. The beam is steered in the direction opposite to the IMS setup for the lateral verification and towards the bottom of the phantom in the vertical dataset. Abbreviation: IMS, imaging and monitoring system.

In the course of CIRT plan delivery, the pencil-beam position is monitored by the SPM. The real-time sensitivity of the fragment vertex distributions to the beam position in the target was similarly studied. Shifts of the beam spot in the phantom were induced by the steering dipole magnets in submillimeter (<0.5-mm) and centimeter-scale increments. The beam was shifted away from the IMS setup for the lateral position verification and towards the bottom of the phantom for the vertical direction. The projected vertex profile along the phantom width (**Figure 3d**) was fitted with a Gaussian to determine the distribution mean (Y_vertex_). The beam position (Y_beam_) along the entrance path before delivery was measured by a virtual detector placed −10 cm upstream of the isocenter. The precision of the beam positional verification Δ_Y_ = Y_beam_ − Y_vertex_ in the lateral (Δ_X_ vertical) direction is shown in **Figure 5** at 220-MeV/u incident energy. The error bars are of statistical origin. A direct linear dependence relates Δ_Y_ to the shifted beam position as shown in **Equation 3**. This dependence holds at intermediate energies as well as for the vertical direction as discussed subsequently.



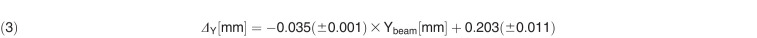




## Discussion

With the promise of higher therapeutic effectiveness and nontarget tissue sparing in CIRT, in vivo monitoring of this emerging modality would increase confidence in dose delivery accuracy for the substantially more destructive carbon ions. The primarily induced lesions in CIRT, double-strand DNA breaks, present marked challenges for the cell's repair system [34]. Real-time verification of the CIRT range would also allow precision gains in single-field treatment techniques that seek either to minimize the irradiated volume [35] or to better monitor target motion [36], compared to conventional multi-field plans. Moreover, online monitoring in CIRT could assist mitigation strategies aiming to protect organs at risk affected by respiratory and other physiological motion.

As shown in **Figure 3**, the reconstructed vertex depth distribution recreates the target phantom boundaries. The vertex depth profile could be used to detect patient mispositioning in real time. The events preceding the phantom entrance arise, in part, from artifacts of the beam back-projection method. To mitigate this artifact, as well as to reduce the detector count-rate, we evaluated an alternative IMS setup as mentioned earlier. This setup used 2 detector stacks operated in coincidence. The setup's total detection efficiency (∼4%), a convolution of the intrastack and interstack coincidence efficiencies, resulted in substantially limited statistics. We concluded that the coincidence IMS's feasibility requires a larger detector aperture (10×10 cm^2^).

For the range verification, the comparably larger uncertainties on certain calibration points (**Figure 4**) stem from poorer fits of the reconstructed vertex depth distributions. Errors on the fit free parameters propagate to the Z50 marker calculation and therefore to the inferred range. The steepness of the vertex distribution fall-off was found to depend on the beam range. The slope of the tangent to the distal fall-off is higher at the proximal regions of the phantom, gradually decreasing with increasing depths. Also, at lower incident beam energies, the fragment energies are lower while they must traverse longer distances to reach the IMS detector. These effects influence the depth profile modeling with its analytical form a priori unknown. The incurred additional interactions alter the shape of the vertex track distribution. While the range verification uncertainties limit the IMS technique's accuracy, this could in principle be improved by investigating a more adapted analytical model or alternatively using numerical fits of the vertex distribution.

For the beam spot in vivo location, at intermediate energies and in the lateral and vertical directions, beam shifts of < 3 cm were verified with an overall accuracy of better than 0.6 mm. At shallower irradiation depths (eg, 4.2-cm range), the precision is limited in average to 0.2 mm (35% error) and the linear dependence observed in **Equation 3** is not sustained. In the vertical direction (X), the vertex profile exhibits sensitivity to multiple scattering undergone by fragments escaping the target. Therefore, the X vertex distribution (**Figure 3d**) deviates from a standalone normal profile, which reflects in larger uncertainties on Δ_X_ (**Figure 5b**) compared to the lateral direction. Despite this, the deduced spot shift linear relation (**Equation 3**) persists with quite comparable slopes (−0.02 to −0.03). These results support that the beam position in the CTV could be inferred with relatively good precision. However, the finite single IMS detector aperture limits only fragments incoming within that acceptance to be detected.

The present proof-of-concept study has several limitations. Particle beams manifest a distinct sensitivity to changes in geometry and tissue density. Hence, an apparent clinical limitation is the IMS performance in presence of patient tissue heterogeneities. In this work, the particle beam was made to traverse a bone to soft-tissue–like interface, yielding a range determination accuracy superior to 5 mm. Future investigations are necessary to assess the impact of complex density heterogeneities on the achievable range verification precision. Similarly, an additional future test for the IMS imaging lies in probing range uncertainties for targets exhibiting respiratory motion. We note that the attainable range verification accuracy might also depend on the treated volume size. While the IMS detects the 2D (X, Y) beam position in the phantom, range verifications for large mesh volumes may only be available as averaged over few beam spots.

From the hardware perspective, a stringent limitation of the IMS technique is the achievable detector timing resolution. As noted, the reconstruction algorithm rejected multiplicity (pile-up) events for insufficient precision. The IMS data throughput, on the other hand, is plausibly manageable by modern high-performance digital front-end electronics [37]. Also, readout controls (eg, CMOS, ASIC chips) commonly used in frame mode medical imaging readily allow list-mode acquisition. Laboratory measurements with candidate detector-acquisition systems are ultimately necessary to validate the technique further. In this regard, a scintillator detector coupled to a silicon photomultiplier (SiPM) readout array [38, 39] presents an attractive alternative to a pixelated silicon sensor. In addition to superior timing performance, the SiPM technology offers the suitable ruggedness for long-term clinical practice usage. Finally, the cost-effectiveness of a scintillator-SiPM ensemble makes it possible to mount several IMS detector units at different angles around the treated volume. Similar to tomosynthesis mammography, a C-arm–like reconstruction can be implemented with anticipated improvements, compared to a 1-angle back-projection.

## Conclusions

We reported a nuclear fragmentation imaging conceptual study for a prospective AS-CIRT nozzle using the TOPAS-Geant4 MC simulation framework. Based on a simple detector-acquisition system, basic vertex track reconstruction, and data analysis, the IMS imaging technique yielded promising capabilities for verifying in vivo, in real time, and per scanned spot with at least 4 treatment delivery monitoring features: depth dose profile, beam ion range, beam position, and entrance-exit planes of the irradiated target. The presented technique provides physical proof-of-concept and guidance for future monitoring hardware prototype development efforts.
